# Pleural effusions: seeing transudate–exudate differentiation in a different light

**DOI:** 10.1183/23120541.01362-2025

**Published:** 2026-03-16

**Authors:** David J. Feller-Kopman, Craig A. Mounsey

**Affiliations:** 1 Pulmonary and Critical Care Medicine, Dartmouth Hitchcock Medical Center, Lebanon, NH, USA; 2 Oxford Pleural Unit, Oxford University Hospitals, Oxford, UK

## Abstract

Pleural effusions are a common medical presentation, with an estimated incidence of 337 per 100 000 of the population [1]. The causes of pleural effusion are diverse and making an accurate diagnosis is therefore paramount. A typical first step in the evaluation of patients with pleural effusion is to differentiate the effusion as a transudate or exudate [2]. Transudates are a result of systemic factors leading to increased capillary hydrostatic pressure or reduced oncotic pressure, while exudates reflect an inflamed pleura with causes including malignancy, infection and autoimmune conditions.

## Shareable abstract

**The “triple combination” test developed by Porcel and colleagues avoids the need for simultaneous serum sampling and has similar diagnostic performance characteristics to Light's criteria** https://bit.ly/4o5jrp7

Pleural effusions are a common medical presentation, with an estimated incidence of 337 per 100 000 of the population [1]. The causes of pleural effusion are diverse and making an accurate diagnosis is therefore paramount. A typical first step in the evaluation of patients with pleural effusion is to differentiate the effusion as a transudate or exudate [2]. Transudates are a result of systemic factors leading to increased capillary hydrostatic pressure or reduced oncotic pressure, while exudates reflect an inflamed pleura with causes including malignancy, infection and autoimmune conditions.

Light's criteria, first published in 1972, remarkably, remain the diagnostic reference standard for exudate–transudate differentiation [3]. While it has been consistently demonstrated that Light's criteria misclassify ∼25% of transudates as exudates [4], and the approach requires simultaneous pleural fluid and serum sampling, multiple previous attempts to develop alternative strategies have failed to show an increased diagnostic utility.

In this issue of *ERJ Open Research*, Porcel *et al*. [5] present the most convincing challenger to Light's criteria to date. The authors used a sample of >7000 patients with pleural effusion diagnosed at their hospital between January 1994 and March 2025, divided into derivation (n=5000) and validation (n=2280) cohorts. Having confirmed the levels of pleural fluid parameters achieving the optimal diagnostic performance in the derivation cohort, the authors used their validation cohort to compare the performance of their “triple combination” test (defined as pleural fluid protein >3 g·dL^−1^, lactate dehydrogenase (LDH) >250 IU·L^−1^ (more than two-thirds of their laboratory's upper limit of normal serum LDH) or cholesterol >55 mg·dL^−1^) and a “double combination” test (pleural fluid LDH and cholesterol) against Light's criteria. While the double combination demonstrated limited sensitivity for identifying exudates, the triple combination showed similar performance characteristics to Light's criteria, with a sensitivity of 98%, specificity of 70% and area under the curve of 0.834 for correctly identifying exudates in their validation cohort. The authors then used McNemar's test to rigorously compare the triple combination against Light's criteria. The triple combination test classified significantly fewer exudates than Light's criteria in the derivation cohort (103 *versus* 54 false negatives, p<0.001); however, no significant difference was observed in the validation cohort (50 *versus* 38 false negatives, p=0.241). Conversely, the triple combination correctly reclassified a number of transudates labelled as exudates by Light's criteria (20% in the derivation cohort and 19% in the validation cohort). Only 11% and 14% of transudates were better classified by Light's criteria compared to the triple combination in the derivation and validation cohorts, respectively.

The key question arising from this work is whether the authors' triple combination test is a significant improvement on Light's criteria, to the extent that it should be used instead of or in addition to Light's criteria. While the authors acknowledge that many patients with pleural effusion will undergo blood testing, they argue that avoiding the need for simultaneous blood and pleural fluid sampling is an advantage, given that serum LDH is often omitted in routine clinical practice, blood results can be delayed (such as with weekend procedures), and blood sampling can be technically difficult and is often not performed in outpatient settings. One caveat that readers should consider is the generalisability to other centres. The upper limit of normal for LDH in the authors' laboratory was 378 IU·L^−1^, resulting in a two-thirds upper limit of normal for pleural fluid of 250 IU·L^−1^. However, this number varies across laboratories (*e.g.* in one of our laboratories, the upper limit of normal for serum LDH is 202 IU·L^−1^). Furthermore, it does appear that the triple combination test is superior to Light's criteria in terms of reducing the number of false-positive exudate results. This would certainly be an advantage of the test, given that incorrectly identifying effusions as exudates can cause patient harm through unnecessary further invasive investigations. However, arguably, the core tenet of the initial approach to transudate–exudate differentiation should be to avoid false negatives, in which exudates are classified as transudate, as this can lead to delays in the diagnosis of individuals with potentially critical pathologies. It is clear in this large cohort of patients that Light's criteria continue to perform extremely well in this regard. While there was no significant difference in the false-negative rates between the triple combination and Light's criteria in the validation cohort, it is concerning that in the larger derivation cohort, the triple combination test resulted in a significantly higher rate of false negatives.

With the above in mind, it will be interesting to see whether the triple combination finds a role in the diagnostic process for pleural effusions. We know of few other tests that have stood the “test of time” as well as Light's criteria, and they have become deeply ingrained in both clinical practice and medical education. Porcel *et al*. [5] offer a new way to identify transudates and exudates without relying on serum samples, and with an apparent improvement in the correct identification of transudates. However, whether these will be viewed by clinicians as advantages of sufficient importance for routine use, especially given the caveat of a potential increase in the false-negative rates for exudate diagnoses, remains to be seen.
